# Behavioural responses of *Anopheles gambiae *sensu stricto M and S molecular form larvae to an aquatic predator in Burkina Faso

**DOI:** 10.1186/1756-3305-5-65

**Published:** 2012-03-31

**Authors:** Geoffrey Gimonneau, Marco Pombi, Roch K Dabiré, Abdoulaye Diabaté, Serge Morand, Frédéric Simard

**Affiliations:** 1 Institut de Recherche pour le Développement (IRD), UMR IRD224-CNRS 5290-Université de Montpellier 1-Université de Montpellier 2 MIVEGEC (Maladies Infectieuses et Vecteurs: Ecologie, Genetique, Evolution et Contrôle), team BEES (Biology, Ecology and Evolution of vector Systems), 911 Avenue Agropolis, Montpellier, BP 64501 34394, France; 2 Institut de Recherche en Sciences de la Santé (IRSS), Direction Régionale de l'Ouest (DRO), 399 Avenue de la Liberté, Bobo-Dioulasso 01 BP 545, Burkina Faso; 3 Sezione di Parassitologia, Dipartimento di Sanità Pubblica e Malattie Infettive, Università di Roma "Sapienza", P.le Aldo Moro 5, 00185 Roma, Italy. Institut Pasteur - Fondazione Cenci Bolognetti, P.le Aldo Moro 5, Roma 00185, Italy; 4 Institut des Sciences de l'Evolution de Montpellier (ISEM), UMR 5554-Centre National de la Recherche Scientifique (CNRS)-Institut de Recherche pour le Développement (IRD)-Université de Montpellier 2, Université de Montpellier 2, Montpellier CC 065, 34095, France

## Abstract

**Background:**

Predation of aquatic immature stages has been identified as a major evolutionary force driving habitat segregation and niche partitioning in the malaria mosquito *Anopheles gambiae sensu stricto *in the humid savannahs of Burkina Faso, West Africa. Here, we explored behavioural responses to the presence of a predator in wild populations of the M and S molecular forms of *An. gambiae *that typically breed in permanent (e.g., rice field paddies) and temporary (e.g., road ruts) water collections.

**Methods:**

Larvae used in these experiments were obtained from eggs laid by wild female *An. gambiae *collected from two localities in south-western Burkina Faso during the 2008 rainy season. Single larvae were observed in an experimental arena, and behavioural traits were recorded and quantified a) in the absence of a predator and b) in the presence of a widespread mosquito predator, the backswimmer *Anisops jaczewskii*. Differences in the proportion of time allocated to each behaviour were assessed using Principal Component Analysis and Multivariate Analysis of Variance.

**Results:**

The behaviour of M and S form larvae was found to differ significantly; although both forms mainly foraged at the water surface, spending 60-90% of their time filtering water at the surface or along the wall of the container, M form larvae spent on average significantly more time browsing at the bottom of the container than S form larvae (4.5 vs. 1.3% of their overall time, respectively; *P *< 0.05). In the presence of a predator, larvae of both forms modified their behaviour, spending significantly more time resting along the container wall (*P *< 0.001). This change in behaviour was at least twice as great in the M form (from 38.6 to 66.6% of the time at the wall in the absence and presence of the predator, respectively) than in the S form (from 48.3 to 64.1%). Thrashing at the water surface exposed larvae to a significantly greater risk of predation by the notonectid (*P *< 0.01), whereas predation occurred significantly less often when larvae were at the container wall (*P *< 0.05) and might reflect predator vigilance.

**Conclusions:**

Behavioural differences between larvae of the M and S form of *An. gambiae *in response to an acute predation risk is likely to be a reflection of different trade-offs between foraging and predator vigilance that might be of adaptive value in contrasting aquatic ecosystems. Future studies should explore the relevance of these findings under the wide range of natural settings where both forms co-exist in Africa.

## Background

Predation is a selective force that shapes the behaviour of species, their population sizes and community structures [1,2], including those of aquatic communities [3,4]. Flexible behavioural repertoires allow prey species to adopt risk-reducing behaviours when high predation risks are detected, thereby minimising negative fitness trade-offs between foraging activity and predator avoidance [5-7]. As a result, species that exhibit plastic behaviour in response to predation might be more successful in colonizing new or altered environments that pose greater predation risks [8]. Furthermore, adaptations to predation may play a pivotal role during ecological speciation by prompting adaptive trait divergence directly among lineages, and hence drive diversification and radiation [9-11].

In West Africa, the malaria mosquito *Anopheles gambiae sensu stricto *(Diptera: Culicidae) offers a compelling opportunity to study the impact of predation on population structure and distribution due to the wide variety of ecological contexts they inhabit in relation to predation pressure. *Anopheles gambiae s.s*. (hereafter referred to as *An. gambiae*) has split into two genetically differentiated 'molecular forms' provisionally named M and S [12,13], among which gene flow appears to be highly restricted in at least parts of their overlapping distributions [14-20]. These molecular forms are widely sympatric throughout West Africa and share many behavioural and ecological features, such as adult host feeding behaviour and resting site preferences. Their larvae typically develop in temporary, rain-dependant freshwater aquatic habitats (e.g., puddles, road ruts and quarries) and larval development sites are extensively shared throughout their common distribution range [21,22]. However, M form larvae also thrive in permanent freshwater habitats, such as rice irrigation schemes, whereas S form larvae do not develop successfully in such habitats [22,23]. Costantini *et al. *[17] further demonstrated that this finding is consistent with a recent niche expansion of the M form into marginal habitats in Burkina Faso. Moreover, predation of larvae has been highlighted as a major force prompting niche differentiation between these incipient mosquito species in Burkina Faso, where, it has been proposed, heterogeneities in behavioural responses to predators are phenotypic traits that have led to segregation in M and S form populations [22,24,25].

In western Burkina Faso, the main predator of mosquito larvae in freshwater habitats is the backswimmer, *Anisops jaczewskii *Hutchinson 1928 (Hemiptera: Notonectidae) [26]. Notonectids are widespread insect predators that have been shown to act as an important organizer of aquatic invertebrate community structure, in that they significantly reduce, and sometimes eliminate, larger pelagic or neustonic species [2] such as mosquito larvae [27,28]. We have previously used this mosquito predator to experimentally challenge *An. gambiae *M and S form larvae in south-western Burkina Faso and have shown that S form larvae suffer higher predation rates than M form larvae, suggesting increased predator avoidance in the latter [24]. Here, we specifically compared the nature and extent of differences in predator-induced behaviour in M and S larvae exposed to acute predation risk. We also investigated whether some behavioural traits (activities or locations occupied) entail greater risks of predation by *A. jaczewskii *than others.

## Methods

### Mosquito source

Larvae used in these experiments were obtained from eggs laid by wild female *An. gambiae *collected from two localities in which both molecular forms were sympatric in south-western Burkina Faso during the 2008 rainy season. M form *An. gambiae *females were collected in the village of Bama (11°23'14"N, 4°24'42"W). The village is surrounded by a 1,200 ha irrigated rice field area where the M form predominates in collections of adult mosquitoes throughout the year (> 95%) [29,30]. S form females could not be collected in sufficient number in Bama at the time of the experiment. These were collected 50 km south-east of Bama in Soumousso (11°00'46"N, 4°02'45"W), a village within the typical Guinean savannah habitat of the area, where the S form is dominant during the rainy season [31,32]. Wild gravid and/or blood-fed females collected indoors in Bama and Soumousso were placed individually in oviposition cups and maintained under standard insectary conditions (28 ± 1°C, 80 ± 10% RH and 12-12 L:D) with permanent access to 5% glucose solution. After oviposition, females were placed individually in tubes containing a desiccant and species and molecular form was assessed by PCR performed on a single leg [33]. Newly hatched larvae were pooled according to their molecular form and reared in insectary pans at a density of 0.5 larva/cm^2^. Daily, larvae were fed *ad libitum *with TetraMin^® ^Baby Fish food. They were starved for 24 hours prior to the experiments to standardize hunger levels.

### Predator source

*Anisops jaczewskii *is a widespread aquatic predator found in temporary as well as permanent aquatic habitats in Burkina Faso [25]. For logistical reasons, the predators were collected in the rice field irrigation canals in the village of Bama, where the species was previously found to be abundant [25]. Predators were caught using a plastic bowl and transferred to bottles for transportation to the insectary in Bobo-Dioulasso. They were subsequently placed in individual plastic cups to avoid cannibalism [34]. Late 4^th ^and 5^th ^instars juveniles were used and they were starved for 48 hours prior to the experiments.

### Behaviour of *Anopheles gambiae *larvae

Instantaneous scan samples were used to quantify larval behaviour [35]. Larvae of each molecular form were placed individually into 400 mL circular plastic cups (11.4 cm in diameter) filled with 200 mL of spring water and observations were conducted between 8:00 AM and 12:00 AM every day, under controlled ambient conditions (28 ± 1°C, 80 ± 10% RH) and at day light. After five minutes acclimation, activity and location of the larva within the container were recorded at a time-interval of 1 minute during a period of 30 minutes (i.e. 30 scan samples per larva). The whole process was repeated for twenty specimens (i.e. biological replicates) per larval instar for each molecular form. First instars were not used because of their small size, which did not allow for some behavioural traits to be determined precisely. In total, 60 larvae of each molecular form were observed.

As no larval ethogram exists for *An. gambiae*, a behavioural inventory was devised prior to behaviour quantification (see Additional File 1). According to Juliano and Reminger [36], four major activities can be reliably identified: 1) Resting: larva not feeding and not moving through the water; 2) Filtering: larva filters at the water surface with mouthparts, but no body movement (although in open water, the movement of mouthparts leads to drifting of the larva); 3) Browsing: larva underwater moves along the surfaces of the container, working mouthparts against the surface, presumably scraping food; and 4) Thrashing: larva moves through the water propelled by vigorous lateral movements of the whole body, which results in a reverse movement. Furthermore, the four locations within the container included: 1) Surface: larva located at the water surface, terminal spiracle in contact with the air-water interface; 2) Wall: larva in contact or < 2 mm away from with the container wall; 3) Bottom: larva in contact or < 2 mm away from the container bottom; 4) Middle: larva > 2 mm away from the water surface, the wall, and the bottom.

The proportion of time spent in each activity or location was estimated for each test larva by the proportion of observations in that activity or location [35]. To reduce the number of variables and to obtain uncorrelated descriptors of behavioural patterns, the mean proportions of activities and locations were analysed by Principal Component Analysis (PCA). Principal components (PCs) with eigenvalues > 1 were retained [37] and PCs scores were analysed by a multivariate analysis of variance (MANOVA), with PCs scores as response variables and Form (i.e. M or S), Instars (i.e. 2^nd^, 3^rd ^and 4^th^) and their interaction (i.e. Form x Instars) as model effects. Standardized Canonical Coefficients (SCCs) were used to interpret the relative contribution of PCs to significant effects [38]. Statistical analyses were performed with the R software [39].

### Behavioural response to presence of a predator

Activities and locations of *An. gambiae *larvae were recorded in the absence and in the presence of the predator, *A. jaczewskii*. Trials were carried out in 400 mL circular plastic cups (11.4 cm in diameter) filled with 200 mL of spring water, between 8:00 AM and 12:00 AM every day, under controlled ambient conditions (28 ± 1°C, 80 ± 10% RH) and at day light. One replicate with and one without a predator were conducted at the same time with larvae from the same population (*i.e*., molecular form) and age class. The entire process was replicated at least 20 times per larval instar and per molecular form. One specimen of *A. jaczewskii *was added to the 'treatment' plastic cup and constrained using an open-ended transparent plastic tube placed vertically in the cup [24,40]. An empty tube was placed in the 'control' cup. One *An. gambiae *(M or S) larva was introduced into the cup and, after a 5 min acclimation period, the tubes were slowly withdrawn, releasing the predator into the 'treatment' cup. Because predation ended some trials quickly, larva activity and location were recorded every 15 seconds for 7.5 min, for a maximum of 30 observations in paired control and treatment cups.

As in the previous experiment, proportions of the different activities and locations were analysed using PCA and MANOVA, with PCs (with eigenvalues > 1) scores as response variables and Form (i.e. M or S), Instars (i.e. 2^nd^, 3^rd ^and 4^th^), Predator (i.e. presence or absence) and all second and third order interactions as effects. SCCs were used to interpret the relative contribution of PCs to significant effects. Trials with less than 12 observations (i.e. when larvae were captured within a 3-min period) were excluded from the analysis to reduce errors inherent in proportions based on very low sample sizes [36].

### Predation and risky behaviours

To assess if particular behaviours lead to a greater risk of predation than others, we compared the activities and locations observed immediately before capture with those observed for larvae exposed to *A. jaczewskii*, but not captured at the same time [36]. Notonectids detect their prey using visual stimuli and/or mechanosensory reception [41,42]. Movement, therefore, in addition to increasing encounter rate, increases predation risk. Data from the previous experiment were used in this analysis. For one larva captured by the predator at a time *t *(n = 51), we compared the behaviour of larvae not captured at the same time (n = 459) in other replicates in the presence of *A. jaczewskii*. If we suppose that all activities and locations are equally risky and that molecular forms move at the same speed, significant differences between the 'capture' and 'no capture group' will highlight variation in behaviours associated with higher risks of predation. By this approach, we did not separate 'attempt to escape the predator' from the behaviour that revealed the larvae to the predator, because both would eventually lead to prey capture. The proportion of time spent in each activity and location was compared between groups (capture, no capture) using nonparametric Wilcoxon tests.

## Results

### Behaviour of *Anopheles gambiae *molecular forms

**Figure 1:**
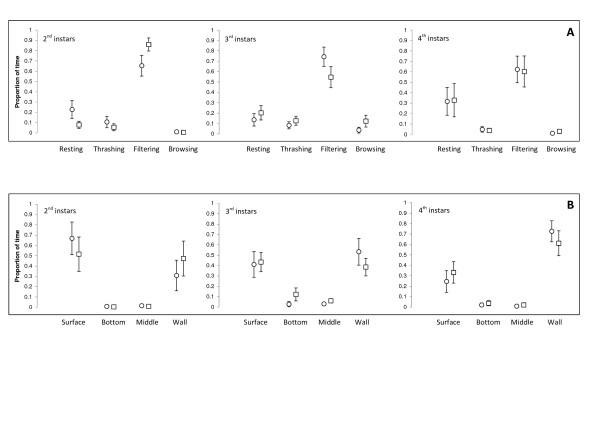
**Measured relative proportions (mean ± 95% confidence interval) of time spent in different activities (A) and locations (B) for larvae of *An. gambiae *M (squares) and S (circles) form across three larval instars in the absence of a predator**. Proportions of the four activities and locations add up to 100% within each form and instars, respectively.

**Figure 2:**
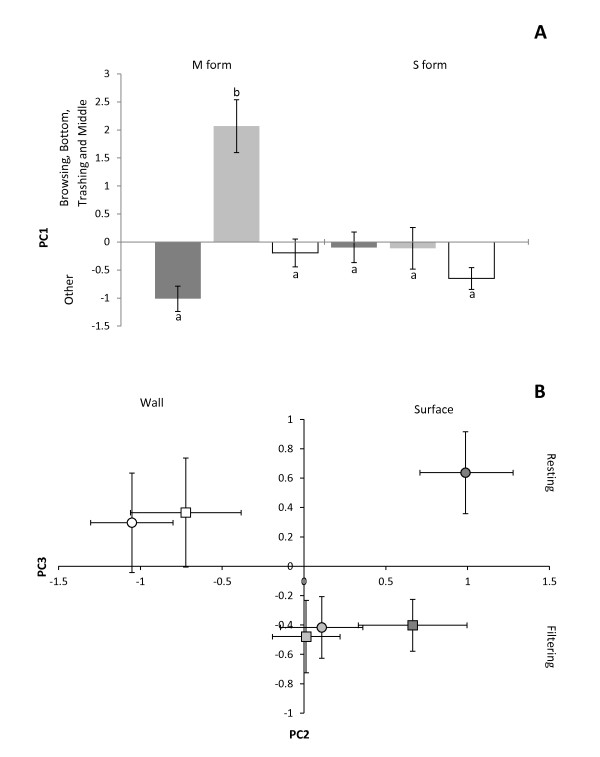
**Principal Component Analysis (PCA) of larval behaviours in the M and S forms of *An. gambiae *in the absence of a predator**. **A) **Principal Component 1 (PC1) shows the relationship between time spent browsing at the bottom of the container and thrashing in the middle *vs*. all other behaviours of 2^nd ^(dark grey), 3^rd ^(light grey) and 4^th ^(white) instar larvae of *An. gambiae *M and S molecular forms. Mean (± SE) with similar letters are not significantly different from one another (Tukey-Kramer multiple comparisons test). **B)** Biplot along PC2 and PC3 (mean ± SE) showing the behaviour of 2^nd ^(dark grey), 3^rd ^(light grey) and 4^th ^(white) instars larvae of *An. gambiae *M (squares) and S (circles) molecular forms.

Throughout the observation period, larvae of the M and S form of *An. gambiae *spent most of their time filtering water at the surface or at the wall of the container (60-90% of their time), depending on the instars (Figure 1). Table 1 shows the results of the Principal Component Analysis (PCA) and interpretation of the three PCs (with eigenvalues > 1) accounting for 81.4% of the variation in larval behaviour. Subsequent MANOVA (Table 2) indicated that the behaviour differed between forms and between instars, with SCCs highlighting that PC1 (frequent browsing at the bottom and thrashing in the middle) contributed most to all significant effects. Indeed, larvae of the M form browsed at the bottom and thrashed in the middle significantly more than the S form (4.5% vs. 1.3% of their overall time, respectively; Tukey-Kramer multiple comparisons test: *P *= 0.026) with 3^rd ^instars of the M form spending up to 12% of their time browsing at the bottom of the container (Figure 1, Figure 2A). In both the M and S forms, fourth instar larvae were less active than earlier instars and rested more at the wall of the container (32.2% for 4^th ^instars vs. 17.0% and 15.3% for 3^rd ^and 2^nd ^instars, respectively) (Figure 1, Figure 2B).

**Table 1:** Rotated factor patterns testing for behavioural differences between larval instars (2^nd^, 3^rd ^and 4^th^) of *An. gambiae *M and S molecular forms

	Principal component (eigenvalue)		
**Variables**	**PC1 (2.81)**	**PC2 (2.02)**	**PC3 (1.67)**

Bottom	**0.84**	-0.11	0.02
Browsing	**0.89**	-0.1	0.03
Filtering	-0.35	-0.02	**-0.93**
Middle	**0.76**	0.13	0.08
Resting	-0.13	-0.06	**0.98**
Surface	-0.11	**0.98**	-0.03
Thrashing	**0.62**	0.3	0.09
Wall	-0.19	**-0.96**	0.02

Values greater than 0.40 (in bold) represent strong factor loading contributions for each principal component [43]. High positive scores on PC1 indicated that larvae allocated more time to browsing at the bottom of the container and thrashing in the middle compared to other behaviours. A large positive score on PC2 indicated that larvae spent more time at the surface and a negative score indicated more time spent at the wall. A high positive score on PC3 indicated more time spent resting as opposed to filtering

**Table 2:** MANOVA for principal components (PCs) of the behaviour of larvae (2^nd^, 3^rd ^and 4^th ^instars) of the *Anopheles gambiae *M and S molecular forms

				Standardized canonical coefficients			
**Variables**	**Num. d.f**.	**Den. d.f**.	**Pillai's trace**	***P***	**PC1**	**PC2**	**PC3**

Forms	3	112	0.066	0.054	**-0.847**	-0.021	-0.608
Instars	6	226	0.510	< 0.001	**-0.701**	0.587	-0.498
Forms-Instars	6	226	0.214	< 0.001	**0.939**	-0.083	-0.262

PCs contributing strongly to significant effects are shown in bold

### Behavioural response to predation

**Figure 3:**
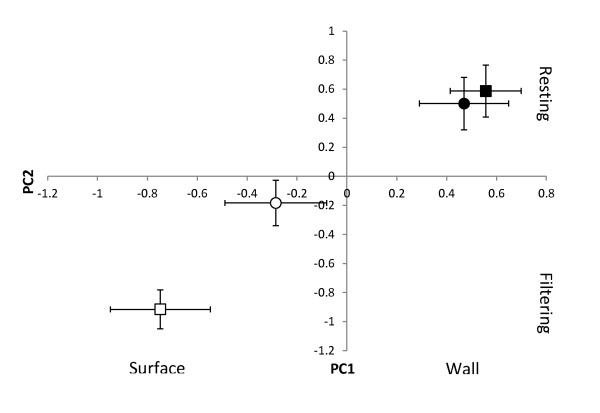
Biplot along Principal Component 1 (PC1) and PC2 (mean ± SE) showing the behaviour of *An. gambiae *larvae of the M (squares) and S (circles) forms without (empty symbols) and in the presence of the predator, *A. jaczewskii *(filled symbols)

PCA and MANOVA analysis indicate that the presence of the predator modified the behaviour of both molecular forms in a similar way, although to a different extent in the two forms. Three PCs with eigenvalues > 1 accounted for 73.1% of the variation in behaviour (Table 3) and SCCs indicated a major effect of PC1 (wall vs. surface) and PC2 (resting vs. filtering) to all significant effects (Table 4). Accordingly, larvae of the M and S forms responded in the same way to the physical presence of the predator, resting more at the container wall (Figure 3). S form larvae spent on average 48.3% (95%CI = [39.7%-56.8%]) of the time overall at the wall when there was no predator, and 64.1% (95%CI = [55.6%-72.7%]) of the time when the predator was present (Tukey-Kramer HSD: $\bar{\text{x}}$ = 0.68, P = 0.009), i.e., an increase of 15.8%. M form larvae spent on average 38.6% (95%CI = [31.2%-45.9%]) of the time at the wall when there was no predator, and 66.6% (95%CI = [59.5%-73.6%]) when the predator was present (Tukey-Kramer HSD: $\bar{\text{x}}$ = 1.5, *P *< 0.001), i.e. an increase of 28%, indicating that the behavioural response was twice as pronounced in the M form as it was in the S form.

**Table 3:** Rotated factor patterns testing behavioural responses of *Anopheles gambiae *M and S larval instars (2^nd^, 3^rd ^and 4^th^) to the presence of the predator, *A. jaczewskii*

	Principal component (eigenvalue)		
**Variables**	**PC1 (2.34)**	**PC2 (1.99)**	**PC3 (1.51)**

Bottom	-0.16	0.22	-0.11
Browsing	0.05	-0.18	**-0.60**
Filtering	0.01	**-0.96**	0.15
Middle	-0.07	0.11	**-0.81**
Resting	0.03	**0.96**	0.20
Surface	**-0.97**	-0.05	0.07
Thrashing	-0.14	0.03	**-0.86**
Wall	**0.98**	-0.12	0.15
Interpretation	Surface *vs*. wall	Resting *vs*. filtering	Thrashing, middle and browsing *vs*. other

Values greater than 0.40 (in bold) represent strong factor loading contributions for each principal component (PC). A large positive score on PC1 indicates that larvae spent more time at the wall of the container and a large negative score indicates that it spent more time at the surface. PC2 indicates that more time was spent resting as opposed to filtering. Negative scores on PC3 indicated that larvae allocated more time to thrash in the middle and to browse as opposed to other behaviours

**Table 4:** MANOVA for larval (2^nd^, 3^rd ^and 4^th ^instars) behavioural principal components (PCs) of the M and S forms of *Anopheles gambiae *in response to the physical presence of the predator, *A.jaczewskii*

				Standardized canonical coefficients			
**Variables**	**Num. d.f**.	**Den. d.f**.	**Pillai's trace**	***P***	**PC1**	**PC2**	**PC3**

Forms	3	234	0.039	0.024	0.505	**-0.803**	0.623
Instars	6	470	0.099	< 0.001	-0.160	0.508	**-0.925**
Predators	3	234	0.304	< 0.001	**0.733**	**-0.835**	0.258
Forms-Instars	6	470	0.080	0.004	-0.482	**0.920**	-0.445
Forms-Predators	3	234	0.048	0.009	0.634	**-0.889**	0.325
Instars-Predators	6	470	0.026	0.393			
Forms-Instars-Predators	6	470	0.014	0.757			

PCs contributing strongly to significant effects are shown in bold

### Riskiness of different behaviours

#### Activity

Observation of larvae browsing was rare in this experiment, especially in the S form. We therefore pooled filtering and browsing categories for the analysis in order to eliminate these zero frequencies.

**Figure 4:**
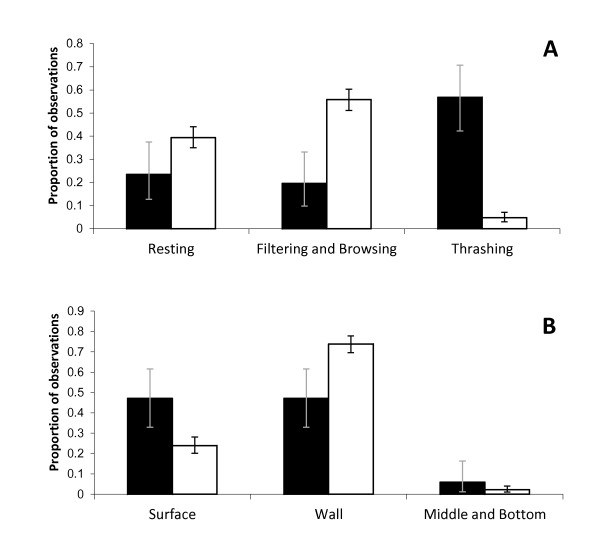
Proportions (mean ± 95% confidence interval) of activities (A) and locations (B) occupied by *An. gambiae *larvae exposed to *A. jaczewskii *in the capture (black columns; which add up to 100% for A and for B) and no-capture (white columns; which add up to 100% for A and for B) groups

Proportions of each activity differed significantly between the capture and no capture groups. Thrashing was significantly over-represented in the capture group (Wilcoxon test, *P *= 0.004) whereas resting (Wilcoxon test, *P *= 0.03) and filtering (Wilcoxon test, *P *= 0.002) were significantly over-represented in the no capture group (Figure 4A).

#### Location

Observation of larvae at the bottom of the container was rare in this experiment. Therefore, we pooled bottom and middle categories for the analysis in order to eliminate these zero frequencies.

Proportions of each location occupied differed significantly between the capture and no capture groups. Wilcoxon tests highlighted that the proportion of observations at the surface and at the wall differed between groups. Observations at the surface were significantly over-represented in the capture group (*P *= 0.016) whereas observations at the wall were most frequent in the no capture group (*P *= 0.013, Figure 4B).

Altogether, these results suggest that thrashing at the water surface entailed the greatest risk of predation by the predator, whereas being at the container wall was the least risky position and might reflect predator vigilance.

## Discussion

This study revealed new and important phenotypic differences in the larval behaviour of wild mosquito populations representative of the M and S molecular forms of *An. gambiae *in western Burkina Faso. Without the presence of a predator, larvae of the S form typically behaved as surface feeders, mainly thrashing at the water surface and foraging through interfacial filtering, while M form larvae spent a significantly greater proportion of time browsing at the bottom of the container and diving more frequently than the S form. In both molecular forms, earlier larval instars were significantly more active than later instars, which allocated more time to resting. Both forms responded to the physical presence of a notonectid predator by modifying their behaviour, spending a significantly greater proportion of time resting at the container wall, although the extent of this shift towards low-risk behaviour was twice as great in the M as it was in the S form. Such differences in the behaviour of molecular forms and their respective levels of behavioural plasticity probably reflect historic differences in the selective pressures on trade-offs between foraging and predator vigilance/avoidance that might be of significant adaptive value [5,7].

In the area of Bama, M form larvae are frequently found in permanent aquatic habitats such as rice paddy fields, where they co-exist with *Culex sp *mosquito larvae [22,23,25]. *Culex *larvae typically feed by browsing at the bottom of such permanent aquatic habitats, where there is a continuous cycle of leaf litter decomposition sustained by intensive bacterial growth [44]. It has been argued that such 'particulate organic matter' found at the bottom of permanent aquatic habitats are a major and highly rewarding food resource for mosquito larvae and other aquatic insects, because particles are coated and sometimes infiltrated with microorganisms of high nutritive value [45]. The higher frequency of browsing at the bottom we observed for the M form larvae might, therefore, provide fitness advantage when colonizing permanent water bodies, exploiting new opportunities for efficient foraging [45,46]. However, because larvae were starved for 24 h prior to observation and no food was added in our experiment (although some resources might have been available through the spring water we used), it is possible that such browsing behaviour might be heightened due to hunger [5,7]. Therefore, the relevance of these findings in natural settings and the role of browsing as an optimal foraging strategy for *An. gambiae *M form in permanent aquatic habitats needs to be further assessed.

In both mosquito populations, 2^nd ^and 3^rd ^instars spent significantly more time filtering at the water surface than 4^th ^instars, which spent more time resting in contact with the container's wall. In his study of predation behaviour in *Notonecta undulata*, Streams [47] highlighted that the proportion of encounters resulting in attacks increased with prey size, due in part to an increase in the predator's reactive distance to prey as prey size increases. Since larger mosquito larvae are more susceptible to predation by *N. undulata*, it is likely that the relatively greater amount of time spent at rest in 4^th ^instars has been selected for because it leads to less predation and ultimately enhanced fitness. Moreover, since notonectids detect prey through prey movement, later larger instars may produce higher intensity or amplitude vibrations when they move and are, therefore, more at risk of detection and detectable from greater distances [42]. Furthermore, because the cost of aquatic locomotion is often size dependant [46], it may be more energy efficient for 4^th ^instars to remain less active than younger instars [36], especially as they undergo energetically costly morphological changes as they transform into pupae [45,48].

The experiments reported here show that the M and S forms of *An. gambiae *modify their behaviour to a significant extent in the presence of a natural predator, the backswimmer *A. jaczewskii*, by becoming less active and positioning themselves at the wall of the container, which appears to be the safest location under our experimental settings. Activity reduction in response to increased predation risk has been shown for a number of species, including mosquitoes [36], crayfish [49], tadpole [50] and voles [51], and might, therefore, represent a general mechanism for predator vigilance. In mosquitoes, reduced movement appeared to reduce both encounter rates with and conspicuousness to *Notonecta *[52,53]. These behavioural modifications suggest that mosquitoes are able to detect a predator's presence, through as yet unknown mechanisms which deserve further investigation [24].

Another important aspect of predator-induced behavioural plasticity, which was not investigated in the present study, is microhabitat use. Sih [54,55] has shown that a shift to the habitat edge can reduce the predation rate by notonectid because they primarily forage away from the edge and are less successful in feeding at the edge. This is in agreement with our results, which show that staying at the wall was the least risky location for mosquito larvae. Notonectids are present in temporary water collections such as those preferred by the S form of *An. gambiae*, although at generally much lower densities than in more permanent water collections [25]. S form larvae are able to develop at the shallow edges of temporary pools, with only a thin film of water around them. Preferential use of this part of the habitat by S form larvae might reduce predation risk by notonectids, as well as by other macroinvertebrates such as *Dytiscidae *or *Libellulidae*, and could, therefore, represent a reliable way to escape predation in this ecological context, reducing the need to mount and maintain a costly anti-predator vigilance. Moreover, other behavioural adaptations could have developed in relation to different predator strategies and preferred areas for hunting (e.g., surface, middle or bottom of the habitat). Additional studies are required to better assess the range of behavioural adaptations observed amongst the various molecular and chromosomal forms in the *An. gambiae *complex that reduce their vulnerability to predation pressures in their respective larval environments. Use of refuges provided by vegetation or other kinds of floating debris commonly found in more permanent larval development sites, such as those where the M form was found in Bama [23], may also be an important component of the M form response to enhanced predation risk, as it was shown to be the case for other aquatic species, including mosquitoes [54,55]. Altogether, these limitations to our work prompt further investigation in order to unravel the proximal mechanisms pertaining to habitat segregation between the molecular forms of *An. gambiae *in Burkina Faso.

## Conclusion

We have shown that there are measurable differences in the behavioural response to an acute predation risk between populations of M and S molecular forms of *An. gambiae *larvae in a rice field area of Burkina Faso. Thrashing at the water surface was the most risky behaviour when it comes to predation by the voracious and widespread notonectid. Presence of the predator in an experimental arena shifted the behaviour of *An. gambiae *larvae towards a safer location at the wall of the container, entailing predator vigilance. This behavioural shift was twice as pronounced in the M as it was in the S form, suggesting different trade-offs between foraging and predator vigilance that might be of adaptive value in contrasting aquatic ecosystems. Further studies are required to explore the relevance of these findings under the wide range of natural settings where these molecular forms co-exist in Africa.

## Competing interests

The authors declare that they have no competing interests.

## Authors' contributions

GG and FS conceived and designed the study. FS, RD and AD supervised its implementation. GG conducted the experiments and analysed the data, with support from SM, MP and FS. GG and FS drafted the manuscript, which was critically revised by MP, SM, RD and AD. All authors read and approved the final manuscript.

## Supplementary Material

Additional file 1 **A behavioural inventory for *Anopheles gambiae *larvae**. Click here for file
