# Supplementary material for: Behavioural responses of Anopheles gambiae sensu stricto M and S molecular form larvae to an aquatic predator in Burkina Faso
Source: Parasit Vectors. 2012 Mar 31;5:65. doi: 10.1186/1756-3305-5-65 (PMC3352179; doi:10.1186/1756-3305-5-65)
Supplement: Additional file 1 — A behavioural inventory for Anopheles gambiae larvae. [file 1756-3305-5-65-S1.PDF]

A behavioural inventory for *Anopheles gambiae* larvae

Field-collected wild *Anopheles gambiae* females were allowed to lay their eggs in individual tubes and their progenies were reared under insectary conditions (temperature 28±1°C, 80±10% RH and 12-12 hours L:D). The experimental arena consisted of circular plastic trays (11.4cm diameter) filled with spring water. Observations were conducted on all instars, except first instars larvae due to their small size, which precluded precise observations. Behaviours listed here are subcategories belonging to one of the four main categories described in the accompanying paper: Resting, Filtering, Thrashing and Browsing.

“Resting” includes:

1) Floating/motionless: Larva motionless, lying parallel to the surface of the water, with the stigmatic apparatus attached to the air-water interface. Head in standard position, dorsal face uppermost. Lateral palatal brushes motionless.
2) Fixed/motionless: As above, but due to the forces of water tension larva is pulled towards the edges of the container. As a result, a resting larva appears to be fixed either parallel or perpendicular to the edge of the container.
3) Lurking: At the bottom of the container, the larva is positioned horizontally or slightly curved, remaining in this position for several seconds, motionless. This behaviour is generally a response to the onset of an environmental stimulus, such as a predator attack or other kinds of mechanic disturbance [1].

“Filtering” includes:

1) Floating/interfacial feeding: Larva lying parallel to the water surface, with the stigmatic apparatus attached to the air-water interface. Ventral face of the head uppermost after counter-clockwise rotation of 180° with only lateral palatal brushes in motion. Due to filtering movement, larva drifts across the water's surface.
2) Fixed/interfacial feeding: As above, except larva does not drift across the water's surface. Larva is fixed to the edge of the container due to forces of water tension. Larva either parallel or perpendicular to the edge.
3) Sub-surface feeding: Motionless at the surface, larva bends the anterior part of the body downward leaving its stigmatic apparatus attached to the water's surface. In this position, larva filters the lower part of the water surface.

“Browsing” includes:

1) Sub-surface grazing/Browsing: Generally underwater, the larva brushes the walls and bottom of the container with its mouthparts to ingest food, but the head does not rotate. During the process, the larva is propelled forward due to lateral palatal brushes movement. The larva could be seen browsing the wall or bottom of the containers.

“Thrashing” includes:

1) Wriggle swim: This behaviour is characteristic of larval mosquito’s locomotion. It consists of flexing and unflexing movements of the whole body which result in a backward movement parallel to the surface [2].
2) Dive: Larva falls down passively, using little wriggling movements.
3) Startle dive/Swim: When larva is disturbed by an environmental stimulus, it suddenly dives rapidly downwards using wriggling movements or swims away vigorously.
4) Rise: Larva rises to the water surface propelled by vigorous wriggling movements.
5) Autogroom: At the surface, larva twists into a ' U ' shape to engage in body cleaning activity. Larva works its mouthparts against its body, particularly towards respiratory spiracles and anal papillae present on the abdomen.
6) Stretching (Homostrophic reflex): Parallel to the water surface, the larva shifts from a straight position to an S-shape position which is maintained for several seconds. Alternatively, larva takes this shape on the right and left side of its body.

References

1. Futami K, Sonye G, Akweywa P, Kaneko S, Minakawa N: **Diving Behavior in Anopheles gambiae (Diptera: Culicidae): Avoidance of a Predacious Wolf Spider (Araneae: Lycosidae) in Relation to Life Stage and Water Depth**. *Journal of Medical Entomology* 2008, **45**(6):1050-1056.
2. Strickman D: **Biosystematics of Larval Movement of Central American Mosquitos and Its Use for Field Identification**. *J Am Mosq Control Assoc* 1989, **5**(2):208-218.
